# The Relationship between Experienced Respiratory Symptoms and Health-Related Quality of Life in the Elderly with Chronic Obstructive Pulmonary Disease

**DOI:** 10.1155/2021/5564275

**Published:** 2021-05-13

**Authors:** Daryadokht Masror-Roudsary, Nasrin Fadaee Aghdam, Forough Rafii, Robabe Baha, Mahboobeh Khajeh, Abbas Mardani

**Affiliations:** ^1^School of Nursing and Midwifery, Iran University of Medical Sciences, Tehran, Iran; ^2^School of Nursing and Midwifery, Shahroud University of Medical Sciences, Shahroud, Iran; ^3^Nursing Care Research Center, Iran University of Medical Sciences, Tehran, Iran; ^4^Tehran University of Medical Sciences, Tehran, Iran; ^5^Nursing Care Research Center, Department of Medical Surgical Nursing, School of Nursing and Midwifery, Iran University of Medical Sciences, Tehran, Iran

## Abstract

**Background:**

Chronic obstructive pulmonary disease (COPD) is one of the diseases that usually present at an advanced age. Respiratory symptoms in patients with COPD are the most important for making treatment decisions and understanding the adverse effects on health-related quality of life (HRQoL). This study aimed to investigate HRQoL in elderly patients with COPD and examine the relationship between this in relation to respiratory symptoms experienced by them and their demographic characteristics.

**Methods:**

This is a descriptive, correlational study of elderly patients with COPD who were hospitalized in five different hospitals in an urban area of Iran. A consecutive sampling method was used. Demographic data form, the respiratory symptoms component of St. George's Respiratory Questionnaire (SGRQ), and the Short Form 36 Health Survey Questionnaire (SF-36) were applied for data collection.

**Results:**

The patients (*n* = 217) reported low HRQoL, and this impairment was more observed in the physical component. There was a significant inverse relationship between the experienced respiratory symptoms and physical (*p*=0.03) and mental (*p* < 0.001) components of HRQoL. Moreover, the female gender, the low level of education, the increased duration of the disease, the increased number of hospitalizations during the past year, and the use of two classes of drugs simultaneously were associated with the impaired HRQoL.

**Conclusion:**

Our findings inform healthcare providers about the negative impacts of respiratory symptoms and other related factors on the HRQoL of elderly patients with COPD. Nurses and other healthcare providers should proactively identify respiratory symptoms and design appropriate caring strategies to improve HRQoL among this group.

## 1. Introduction

The world's elderly population is growing, which is anticipated to increase from 15.7 percent in 2020 to 25 percent up to 2030 [1]. Various criteria confirm successful aging, including not having any diseases such as cancer, cardiovascular and respiratory diseases, and diabetes; good physical and cognitive functions; good mental health as having no depressive symptoms; and lack of physical disability such as inability to walk and ability to do physical activity without receiving any support [2]. However, the phenomenon of aging is known as the most important factor in the incidence and progression of chronic diseases [3]. One of the diseases occurring during this period is chronic obstructive pulmonary disease (COPD), which is a chronic and progressive obstruction of the airway leading to breathing disorder [4]. This disease includes emphysema and chronic bronchitis. It is predicted that, by 2030, this disease will be accounted for seven of the top ten leading causes of disease burden [5] and will reach the third leading cause of death worldwide [6]. Moreover, this disease is one of the major causes of mortality associated with high economic and social costs [7].

Primary symptoms of COPD include shortness of breath, cough, increased sputum, wheezing, and chest congestion. Secondary consequences of the disease include sleep disorders, increased anxiety, and depression that are represented by fatigue, anorexia, and weight loss [8]. Moreover, previous studies have shown that respiratory symptoms particularly frequent coughs and shortness of breath are associated with urinary incontinence in elderly patients with COPD [9–11]. Moreover, elderly patients with COPD experience more respiratory symptoms and physical dysfunction with the exacerbation and the increased duration of the disease [12]. Furthermore, exacerbation of the disease can be associated with some other conditions such as sudden cardiac death, which shows that the impacts of COPD are not limited to the respiratory system [13]. Drug treatments in these patients have the main aims of controlling symptoms, reducing the frequency of disease exacerbation, and improving patients' performance and quality of life [13, 14]. However, it can be associated with serious complications such as osteoporosis [15]. Therefore, the need for proper planning for the proper management of these patients is strongly felt.

Undesirable experiences during the elderly life can lead to depression, isolation, substance abuse, and loss of leisure activities and well-being [16]. COPD, its burden, and the consequences of its treatment or nontreatment can greatly affect the life of the elderly [17]. One of the indicators of the disease's burden is health-related quality of life (HRQoL) assessment [18]. HRQoL includes various dimensions such as physical, social, and mental well-being, which are very different for each patient [19]. HRQoL in patients with COPD is closely related to shortness of breath; physical activity disorders; and mental problems such as depression, anxiety, and other psychological disorders [20].

Maintaining a good HRQoL can be known as the main solution to deal with major life changes and problems [16]. Limited studies have examined the quality of life in the elderly with COPD, and the relationship between its symptoms and HRQoL has not been fully investigated in any of the cases yet. Therefore, this study aimed to investigate HRQoL in elderly patients with COPD and examine the relationship between it in relation to respiratory symptoms experienced by them and their demographic characteristics.

## 2. Materials and Methods

### 2.1. Study Design and Participants

This descriptive, correlational study was performed on elderly patients with COPD who were hospitalized to the selected five referral hospitals in an urban area of Iran. The inclusion criteria were the diagnosis of chronic obstructive pulmonary disease recorded in the medical file and age over 60 years. The exclusion criteria included cooccurrence of other infectious lung diseases (according to the diagnosis recorded in the patient's medical file) with COPD and having physically and mentally debilitating diseases.

The required sample size for the current study was estimated to be 210 according to a similar study [13] with 90% test power and 95% confidence level while it was assumed that the correlation coefficient between respiratory symptoms and their HRQoL be at least 0.22 to be statistically significant. The samples were selected using the consecutive sampling method from the patients who were referred to the pulmonary ward of the study hospitals.

### 2.2. Data Collection Tools

Demographic data form, the respiratory symptoms component of St. George's Respiratory Questionnaire (SGRQ), and the Short Form 36 Health Survey Questionnaire (SF-36) were used for data collection.

The demographic data form contained questions about patients' age, gender, marital status, educational level, COPD type, disease duration, number of hospitalizations during the past year, medication type, comorbidity, and smoking history.

The respiratory symptoms component of the SGRQ was used to assess respiratory symptoms in this study. This questionnaire includes 3 components of respiratory symptoms, activities, and impact. The symptoms' component contains 7 questions on frequency and severity of the experienced respiratory symptoms in the last three months, including cough, sputum production, shortness of breath, attacks of wheezing, good days with few respiratory symptoms, wheezing in the morning, and attacks of chest trouble during the last year. In this questionnaire, the symptom score increases with increasing intensity and repetition. Accordingly, a score of zero indicates the absence of respiratory symptoms and 100 indicates the highest severity of the respiratory symptoms experienced. In the study by Al-Shair et al., the reliability of the questionnaire was calculated by evaluating the internal consistency, and Cronbach's alpha coefficient of the symptoms' component was calculated to be 0.77 [21]. In addition, in Iran, its validity and reliability were evaluated, its reliability was evaluated by assessing the internal correlation of the questions, and Cronbach's alpha coefficient was calculated for the symptoms' component equal to 0.76 [22].

The SF-36 was used to assess HRQoL in the present study. This questionnaire is divided into two main components: physical component summary (PCS) and mental component summary (MCS). PCS includes the subscales of physical functioning, role limitations due to physical problems, bodily pain, and general health perceptions. MCS includes the subscales of social functioning, role limitations due to emotional problems, vitality, and perceived mental health. The total score of each subscale is converted to ranges from 0 to 100. The zero score shows the worst condition and 100 indicates the best condition on the scale. The validity and reliability of the English version of this questionnaire have been confirmed by many studies in different populations [23, 24]. Additionally, the reliability of the Persian version of this questionnaire was evaluated by assessing the internal consistency, and Cronbach's alpha coefficient was calculated to be in the range from 0.77 to 0.90 [25].

### 2.3. Data Collection

Questionnaires were completed by the researchers through the interview with elderly patients with COPD who had met the eligibility criteria. The interviews were conducted in a private location of the pulmonary wards of the study hospitals. Participants provided written consent to participate after being informed about the aims of the study. In addition, participants were informed that they could leave the study at any time without penalty.

### 2.4. Ethical Considerations

Ethical approval for this study was obtained from the ethical committee of Tehran University of Medical Sciences under the code of 90/D/130/1317.

### 2.5. Data Analysis

Data were analyzed using SPSS software, version 25. Descriptive statistics including the mean (standard deviations) for continuous variables and frequency (percentage) for categorical variables were applied to summarize the data. The independent samples *t*-test and one-way ANOVA test were used to investigate the relationship between total HRQoL and demographic characteristics. Furthermore, the independent samples *t*-test was applied to investigate the association between HRQoL and respiratory symptoms. Moreover, multivariable linear regression was conducted to investigate the association between gender, number of hospitalizations within the last year, duration of the disease, and experienced respiratory symptoms and HRQoL. A *p* value less than 0.05 was considered as a significance level.

## 3. Results

A total of 217 elderly patients participated in this study, of which 55 patients (25.3%) were women and 162 (74.7%) of them were men. The mean duration of the disease was 7.1 years with a standard deviation of 9.2 years. This mean was significantly higher in female patients (9.4 ± 11.8) compared to men (6.7 ± 7.9) (*p*=0.03). More than three-quarters of patients (*n* = 167) were suffering from both chronic bronchitis and emphysema. More than 63% (*n* = 138) of patients had a history of smoking. Other demographic characteristics are presented in Table 1.

The mean total HRQoL scores of the patients were 41.25 ± 18.2. The mean PCS was 33.9 ± 17.8, which was significantly lower than MCS (49.0 ± 23.6) (*p* < 0.001). Univariate analyses showed that gender and educational level also affected patients' HRQoL (*p*=0.04). In addition, the duration of the disease (*p*=0.01), the number of hospitalizations during the past year (*p*=0.001), and the use of two classes of drugs simultaneously (*p*=0.002) had significantly affected the mean of HRQoL. Other demographic variables did not have any significant effect on the patients' HRQoL (Table 1).

Although there were differences between the effects of each respiratory symptom on the components of quality of life, attacks of wheezing, attacks of chest trouble during last year, and having good days with few respiratory symptoms were found to be significantly associated with both PCS and MCS of the patients' HRQoL (Table 2).

The multivariable linear regression analysis to examine the relationship between gender, number of hospitalizations within the last year, duration of the disease, and experienced respiratory symptoms and HRQoL is shown in Table 3. An inverse significant association was found between the female gender and PCS of HRQoL (*b* = −7.41, *p*=0.004). In addition, there were inverse significant associations between the number of hospitalizations within last year and total HRQoL (*b* = −3.22, *p*=0.003), PCS (*b* = −4.04, *p* < 0.001), and MCS (*b* = −3.06, *p*=0.03). Further, an inverse relationship was found between the duration of the disease and total HRQoL (*b* = −0.27, *p*=0.02), PCS (*b* = −0.24, *p*=0.04), and MCS (*b* = −0.32, *p*=0.04). Finally, there was inverse relationship between experienced respiratory symptoms and total HRQoL (*b* = −0.22, *p*=0.001), PCS (*b* = −0.14, *p*=0.03), and MCS (*b* = −0.33, *p* < 0.001).

## 4. Discussion

This study described HRQoL among elderly patients with COPD and investigated the relationship between their HRQoL in relation to respiratory symptoms experienced by them and their demographic characteristics. The results showed that elderly patients with COPD experienced low HRQoL, and this impairment was more observed in the physical component. Moreover, a significant inverse relationship was found between the experienced respiratory symptoms and physical and mental components of HRQoL.

In this study, participants reported low HRQoL, particularly in the physical dimension. Consistent with our findings, two previous studies showed that elderly patients with COPD suffer from low HRQoL, especially in the physical dimension [26, 27]. In some other studies, old age was one of the predictors of physical activity [12], which was found to be associated with the decreased quality of life in these patients [28, 29]. Elderly patients with COPD have less body strength compared to healthy individuals. Moreover, these patients have lower physical function ability than healthy individuals, which affects their health-related quality of life [30]. Therefore, our findings suggest that healthcare providers should pay more attention to the quality of life in elderly patients with COPD especially in the physical dimension.

In the present study, some factors such as female gender, low level of education, increased duration of the disease, and increased number of hospitalizations during the past year were observed to be associated with the impaired quality of life in elderly patients with COPD. In addition, in the present study, impairment in the physical dimension of quality of life and duration of the disease was significantly higher in women than men. In the study by Vasiljević et al., female gender and disease severity were among the predictors of physical activity [12] that in turn can affect the quality of patients' life. In general, women probably are more disturbed in their duties due to having multiple roles in their lives when exposed to the disease, and therefore their quality of life may be more affected [31, 32]. Additionally, other studies reported a direct association between education [28] and disease duration [29] with quality of life in patients with COPD, which was consistent with the present study. Patients with a low level of education may have less understanding of the disease process, treatment strategies, and caring programs and may not easily adapt to the disease [33]. Therefore, in the process of patients' education, the level of literacy of patients should be considered in order to provide efficient education that helps to improve their quality of life.

Our study findings showed that patients who took two classes of drugs (bronchodilators and corticosteroids) at the same time reported lower quality of life. In patients with COPD, medications including bronchodilators, corticosteroids, and combination therapies are started to control respiratory symptoms, prevent exacerbation, and improve their quality of life. However, it has been shown that a large number of people with COPD sometimes experience respiratory symptoms, despite the chronic use of several medications [34]. Moreover, it was documented that the inhaled corticosteroids are the secondary cause of osteoporosis in elderly patients with COPD [15], which can have a negative impact on their quality of life.

In the present study, there was a significant inverse relationship between the experienced respiratory symptoms and physical and mental components of HRQoL. Similarly, in the study by Moy et al., the presence of shortness of breath was a more important determinant of quality of life compared to the forced expiratory value in one second (FEV1) [35]. In addition, two other studies evidenced that severe respiratory symptoms were associated with poor HRQoL in patients with COPD [34, 36].

In the present study, the experience of attacks of wheezing, attack of chest trouble during last year, and having a good day with few respiratory symptoms was associated with both impaired physical and mental components of HRQoL. In addition, the experience of shortness of breath only was associated with the physical component of HRQoL. Similarly, a prior study found that the experience of frequent coughs and wheezing in the previous 12 months is an important risk factor for the low physical and psychological domain of quality of life among the elderly with respiratory diseases [37]. In addition, it has been revealed that the exacerbation of the disease is associated with the low quality of life of patients with COPD [12]. A prior study has revealed that the most worrying symptom affecting the quality of life was shortness of breath [20]. Shortness of breath and attacks and intolerance to physical activity are distressing and debilitating for patients with COPD, eventually leading to the impaired physical activity of patients and affecting their quality of life [38]. Moreover, in many patients, the prognosis of shortness of breath is associated with an increased physiological response to fear and activation of fear-related areas of the brain. Accordingly, fear increases shortness of breath and avoids physical activity in these patients [39].

Regarding the results of the present study and existing literature, it can be concluded that the physical dimension of quality of life is more impaired in elderly patients with COPD. Due to physical and mental disabilities and lack of adequate social support in old age [40], there are many concerns about the quality of life and providing a dignified life to the elderly [41]. Improving the HRQoL of elderly populations is one of the priorities for the healthcare system worldwide [16]. Previous studies have recommended that using nonpharmacological methods such as counseling-based training programs and breathing exercises may improve the symptoms of the disease, the level of activity tolerance, and the HRQoL in these patients [42]. Light exercises like yoga, which combines movement, controlled breathing, attention, and meditation, can be used in these patients [43]. Yoga affects both physical and mental statuses, and many studies have identified its therapeutic effects on different chronic diseases such as respiratory diseases, diabetes, and high blood pressure [44]. In addition, yoga is associated with an improvement in six-minute walk distance, FEV1, and HRQoL in patients with COPD and is comparable to pulmonary rehabilitation interventions [43].

### 4.1. Limitations

The present study was a cross-sectional study, and statistical correlations might not always show a cause-and-effect relationship. In addition, the samples in the present study were limited to patients living in an urban area of Iran; therefore, the generalization of the findings to other contexts should be performed with precaution.

## 5. Conclusion

Our study identified that elderly patients with COPD have low HRQoL, and this impairment was more observed in the physical component. Moreover, there was a significant inverse relationship between the experience of attacks of wheezing, attack of chest trouble during last year, and having a good day with few respiratory symptoms and physical and mental components of HRQoL. Furthermore, some factors such as female gender, low level of education, increased duration of the disease, increased number of hospitalizations during the past year, and use of two classes of drugs simultaneously were associated with the impaired HRQoL in these patients. Therefore, our findings inform healthcare providers about the negative impacts of respiratory symptoms and other related factors on the HRQoL of elderly patients with COPD. Nurses and other healthcare providers should proactively identify respiratory symptoms and design appropriate caring strategies to improve HRQoL among this group.

## Figures and Tables

**Table 1:** Relationship between demographic characteristics and HRQoL in elderly patients with COPD.

Variables		N (%)	HRQoL	
			Mean ± SD	*p* value
Gender	Male	162 (74.7)	42.6 ± 18.5	0.04^a^
	Female	55 (25.3)	37.1 ± 16.6	

Marital status	Married	175 (80.6)	42.2 ± 18.6	0.12^a^
	Divorced or widow	42 (19.4)	37.4 ± 15.5	

Level of education	None	132 (60.8)	39.3 ± 17.0	0.04^b^
	Primary and middle school	64 (29.5)	42.8 ± 19.0	
	High school and any academic	21 (9.7)	49.0 ± 20.0	

COPD type	Chronic bronchitis	26 (12)	43.9 ± 18.4
	Emphysema	24 (11.1)	32.8 ± 18.2	0.07^b^
	Both	167 (77)	42.1 ± 17.9	

Disease duration	Up to one year	57 (26.3)	45.0 ± 20.0	0.01^b^
	1 to 15 years	131 (60.4)	41.5 ± 17.7	
	More than 15 years	29 (13.4)	32.5 ± 13.5	

Number of hospitalizations in the past year	One	75 (34.6)	46.2 ± 19.4	0.001^b^
	Two	67 (30.9)	42.0 ± 17.3	
	Three	36 (16.6)	39.6 ± 16.0	
	More than 3	39 (18.0)	32.0 ± 15.7	

Medication type	Bronchodilator	120 (55.3)	44.7 ± 18.8	0.002^b^
	Corticosteroid	2 (0.9)	40.39 ± 5.3	
	Both	86 (39.6)	35.9 ± 14.8	

Comorbidity	Yes	111 (51.2)	40.5 ± 19.3	0.5^,a^
	No	106 (48.8)	42.0 ± 17.0	

Smoking history	Yes	138 (63.6)	40.7 ± 17.0	0.57^a^
	No	79 (36.4)	42.2 ± 20.0	

COPD: chronic obstructive pulmonary disease; HRQoL: health-related quality of life. ^a^Independent samples *t*-test. ^b^One-way ANOVA.

**Table 2:** HRQoL and experienced respiratory symptoms in elderly patients with COPD.

Symptoms		Total HRQoL		PCS		MCS	
		Mean ± SD	*p* value^a^	Mean ± SD	*p* value^a^	Mean ± SD	*p* value^a^
Cough	No	38.7 ± 18.7	0.5	30.3 ± 16.1	0.3	49.3 ± 25.3	0.9
	Yes	41.5 ± 18.2		34.3 ± 18.0		48.9 ± 23.5	

Sputum	No	43.3 ± 21.7	0.5	34.07 ± 21.7	0.9	53.9 ± 26.4	0.25
	Yes	41.0 ± 17.6		33.9 ± 17.3		48.3 ± 23.2	

Shortness of breath	No	51.9 ± 28.7	0.15	47.8 ± 32.0	.04	59.5 ± 29.5	0.3
	Yes	41.0 ± 17.7		33.5 ± 17.2		48.7 ± 23.4	

Attacks of wheezing	No	47.6 ± 22.1	0.02	40.6 ± 23.7	0.06	56.0 ± 25.5	0.04
	Yes	40.0 ± 17.1		32.6 ± 16.3		47.7 ± 23.0	

Attacks of chest trouble during last year	No	44.7 ± 17.0	0.03	37.0 ± 17.6	0.04	53.7 ± 22.0	0.03
	Yes	39.3 ± 18.5		32.1 ± 17.8		46.3 ± 24	

Having a good day with few respiratory symptoms	No	39.2 ± 17.4	<0.001	32.5 ± 17.0	0.002	46.1 ± 22.6	<0.001
	Yes	56.4 ± 17.0		44.0 ± 20.7		70.0 ± 20.2	

Wheezing in the morning	No	41.7 ± 18.6	0.4	34.6 ± 18.3	0.2	49.5 ± 24.0	0.6
	Yes	39.4 ± 16.4		31.1 ± 15.7		47.3 ± 22.7	

COPD: chronic obstructive pulmonary disease; HRQoL: health-related quality of life; MCS: mental component summary; PCS: physical component summary. ^a^Independent samples *t*-test.

**Table 3:** Factors affecting the HRQoL in elderly patients with COPD with emphasis on experienced respiratory symptoms.

Factors	Total HRQoL			PCS			MCS		
	b^a^ (SE)	95% CI	*p* value	b (SE)	95% CI	*p* value	b (SE)	95% CI	*p* value
Gender (female)	−4.6 (2.63)	−9.86, 0.46	0.07	−7.41 (2.57)	−12.46, −2.37	0.004	−3.74 (3.47)	−10.55, 3.06	0.2
Number of hospitalizations within last year	−3.22 (1.08)	−5.35, −1.09	0.003	−4.04 (1.06)	−6.12, −1.95	<0.001	−3.06 (1.43)	−5.87, −0.25	0.03
Duration of the disease	−0.27 (0.12)	−0.52, −0.03	0.02	−0.24 (0.12)	−0.46, −0.02	0.04	−0.32 (0.16)	−0.60, −0.04	0.04
Respiratory symptoms	−0.22 (0.06)	−0.35, −0.08	0.001	−0.14 (0.06)	−0.27, −0.01	0.03	−0.33 (0.08)	−0.50, −0.15	<0.001

CI: confidence interval; COPD: chronic obstructive pulmonary disease; HRQoL: health-related quality of life; MCS: mental component summary; PCS: physical component summary; SE: standard error. ^a^Coefficient was obtained according to the multivariable linear regression.

## Data Availability

The data used in the study are available upon reasonable request.
